# How smokers may react to cigarette taxes and price increases in Brazil: data from a national survey

**DOI:** 10.1186/1471-2458-14-327

**Published:** 2014-04-08

**Authors:** Analice Gigliotti, Valeska C Figueiredo, Clarice S Madruga, Ana CPR Marques, Ilana Pinsky, Raul Caetano, Vera Luiza da Costa e Silva, Martin Raw, Ronaldo Laranjeira

**Affiliations:** 1 National Institute for Alcohol and Drug Policies (INPAD), Department of Psychiatry, Federal University of São Paulo, São Paulo, Brazil; 2 Center for Studies on Tobacco and Health, National School of Public Health, Oswaldo Cruz Foundation (Fiocruz), Rio de Janeiro, Brazil; 3 UT Southwestern School of Health Professional & UT School of Public Health, Dallas Regional Campus, Dallas, TX, USA; 4 UK Centre for Tobacco Control Studies, Division of Epidemiology and Public Health, University of Nottingham, Nottingham, UK

**Keywords:** Smoking, Tobacco use, Tobacco control, Prices and taxes

## Abstract

**Background:**

Despite being the third largest tobacco producer in the world, Brazil has developed a comprehensive tobacco control policy that includes a broad restriction on both advertising and smoking in indoor public places, compulsory pictorial warning labels, and a menthol cigarette ban. However, tax and pricing policies have been developed slowly and only very recently were stronger measures implemented. This study investigated the expected responses of smokers to hypothetical price increases in Brazil.

**Methods:**

We analyzed smokers’ responses to hypothetical future price increases according to sociodemographic characteristics and smoking conditions in a multistage sample of Brazilian current cigarette smokers aged ≥14 years (n = 500). Logistic regression analysis was used to examine the relationship between possible responses and different predictors.

**Results:**

In most subgroups investigated, smokers most frequently said they would react to a hypothetical price increase by taking up alternatives that might have a positive impact on health, i.e., they would “try to stop smoking” (52.3%) or “smoke fewer cigarettes” (46.8%). However, a considerable percentage responded that they would use alternatives that would reduce the effect of price increases, such as the same brand with lower cost (48.1%). After controlling for sex age group (14–19, 20–39, 40–59, and ≥60 years), schooling level (≥9 versus ≤9 years), number of cigarettes per day (>20 versus ≤20), and stage of change for smoking cessation (precontemplation, contemplation, and preparation), lower levels of dependence were positively associated with the response “I would try to stop smoking” (odds ratio [OR], 2.19). Young age was associated with “I would decrease the number of cigarettes” (OR, 3.44). A low schooling level was strongly associated with all responses.

**Conclusions:**

Taxes and prices increases have great potential to stimulate cessation or reduction of cigarette consumption further among two important vulnerable populations of smokers in Brazil: young smokers and those of low educational level. The results from the present study also suggest that seeking illegal products may reduce the impact of increased taxes, but does not eliminate it.

## Background

Tax increases and consequently price increases for tobacco products are addressed in article 6 of The World Health Organization (WHO) Framework Convention on Tobacco Control and are considered to be one of the best policies in reducing the demand for tobacco products [1]. Price increases contribute significantly towards reducing consumption, increasing the number of attempts to quit, promoting cessation, and preventing initiation [2-4]. Econometric studies have shown that a 10% increase in tobacco taxes may lead to a 5 to 8% reduction in the prevalence of smoking [5-7]. There is also evidence in the literature that these policies have a particular impact on young people and low-income populations [2,3].

In addition to smokers’ strategies of limiting consumption to cope with higher prices, studies have shown that they also take up alternatives aimed at reducing costs, which may limit the beneficial impact of price and taxes policies [8,9]. For example, they may change their brand of cigarettes for a cheaper one or they may look for places where their preferred brand, even if illicit products, is sold at lower prices. Smokers who engage in cigarette cost reduction behavior present less chance of trying to stop or stopping smoking [10]. Moreover, they are less likely to make successful attempts to quit.

In 2008, 17.2% of the Brazilian population aged 14 years old or older were smokers (approximately 24.6 million people). Most of them were daily smokers (87.8%) and consumed only cigarettes (95.3%) [11]. Despite being the third largest tobacco producer in the world, Brazil, a Latin American continental country, has a comprehensive tobacco control policy. The WHO Framework Convention on Tobacco Control was ratified in November 2005, but some of its important proposals had already been implemented in the country such as a broad restriction on both advertising and smoking in indoor public places, compulsory pictorial warning labels, and banning misleading descriptors [12]. However, tax and pricing policies have been slowly developed [13].

Cigarette prices in Brazil have been oscillating since the 1990s. In 1989, a federal decree entered into force, which stipulated a fixed *ad valorem* tax on manufactured products (named IPI) of 42.5% of the price of cigarettes to consumers [14]. Soon after the increase in IPI between 1990 and 1993, the real price of cigarettes increased by approximately 78% because of a local tobacco industry strategy. This strategy included raising the price above inflation, exporting a huge amount of low-cost products to Paraguay, and encouraging the return of such products to the country through smuggling and thereby reducing the tax burden [14]. During this period, *per capita* cigarette consumption among adults declined 20.5% [15]. This trend of higher prices and reduced *per capita* consumption in the legal market remained until 1998. However, as of 1994, industry estimates showed an increase in consumption of illicit cigarettes [15]. In 1999, in an attempt to reduce the illicit tobacco market, the Brazilian authorities changed IPI’s structure. Taxes were changed from *ad valorem* to specific and applied in four distinct categories. Furthermore, specific taxes were always raised below inflation [14]. As a result, the real price had dropped by 25% in 2006 compared with the maximum of 1993. Although such events might have stimulated tobacco use, consumption levels remained stable probably as a result of nonprice-related tobacco control measures [15]. New taxes and prices rises for tobacco products were implemented in 2007, 2009, and recently, in 2011 following a public health shift in governmental policies [13]. With the 2011 legislation, the total tax burden on cigarettes went up from 60% to 72%, or 81% of the final retail price [13]. In addition, a minimum price policy was instituted that ensured annual increases from 2012 to 2015 [16]. From 2007 to 2011, a declining trend in per capita consumption of cigarettes in the legal market was observed [13]. Additionally, according to the results of a Brazilian telephone surveillance system on noncommunicable disease’s risk factors, the percentage of current smokers in Brazilian state capitals decreased from 15.6% to 12.1% [17]. It should be noted that the fiscal classification of cigarette brands is determined by federal law and cigarettes have similar prices throughout the national territory [18].

The present study investigated how smokers in Brazil react to new price increases by focusing on expected responses to a future hypothetical price increase that has a positive impact on health such as quitting smoking or reducing the demand for cigarettes instead of smokers adopting alternative behaviors such as switching to cheaper brands or buying from the illegal market. Our study also investigated the predictors of these expected behavior patterns. This information is particularly important because to date Brazil does not have any studies with empirical data on smokers’ responses to price increases and their specific impact in reducing the prevalence of smoking.

## Methods

### Subjects

In 2006, the Brazilian National Alcohol Survey was performed by the National Institute for Alcohol and Drug Policies of the Federal University of São Paulo (UNIFESP). The survey used a probabilistic multistage cluster sample design to select 3007 individuals (including smokers and non smokers) aged 14 years and older from the household population in all regions of the country and achieved a response rate of 66.4%. In the total sample, 500 individuals were smokers. The sampling calculation was based on the alcohol abuse and/or dependence using previously known estimations. The smoking prevalence rate is higher than the alcohol problems rates; therefore, we can ensure the sample size is suitable for robust estimations within this subsample. Subjects were interviewed in their households by trained interviewers using a standard questionnaire after obtaining their informed consent. The study was approved by the Research Ethics Committee of the UNIFESP.

### Study variables

#### Demographic and socioeconomic characteristics

This analysis included sex, education (up to nine or nine or more years of schooling), and four age groups (14–19, 20–39, 40–59, or ≥60 years). A binary variable discriminating adolescents and young adults from older age groups was created for the logistic regression analysis (14 to 24 and 25 years or over), given the known vulnerability of younger subgroups to cigarette price-increase measures.

#### Smoking characteristics

Current smokers were defined as those who had smoked at least 100 cigarettes in their lifetime and were currently smoking on the date of the survey. We analyzed nicotine dependence level and smoking frequency because studies have shown that these two variables are strong predictors of smoking cessation [19,20]. The reported time to smoke the first cigarette after waking up was used as an indicator of tobacco dependence [19,21]. Time to smoke the first cigarette after waking up was assessed by the question: “How soon after you wake up do you smoke your first cigarette?” A lower level of nicotine dependence was indicated by smoking more than 30 minutes after waking up, and a higher level of dependence was indicated by smoking within 30 minutes of waking up [19]. A binary variable was created to separate heavy (>20 cigarettes per day) from nonheavy (≤20 cigarettes per day) smokers. A categorical dummy variable was created to indicate the three stages of change for smoking cessation: precontemplation, contemplation, and preparation [22]. Smokers who were not seriously considering quitting within the next 6 months were in the precontemplation stage. Those who were seriously considering quitting within the next 6 months, but were not considering quitting within the next 30 days, or had not made an attempt to quit lasting 24 hours over the past year or both, were in the contemplation stage. Smokers who were planning to quit within the next 30 days and had made a 24-hour attempt to quit during the past year were classified as being in the preparation stage.

#### Expected smoking behavior after a hypothetical change in cigarette prices

To assess the impact of future cigarette prices on smoking behavior, the respondents were asked: “If the price of packs were to increase, what would you do?” The following nonmutually exclusive responses were proposed: smoke fewer cigarettes, switch to a cheaper cigarette brand, look for a cheaper source for your current cigarette brand, or try to quit. For each question respondents could answer “yes”, “no”, “I don’t know”, or refuse to answer.

### Statistical analysis

We estimated the percentages of smokers’ answers to future price increases according to sociodemographic variables. We used Stata SE software (v. 10.0; Stata Corp, College Station, TX, USA) in all analysis [23]. Logistic regression analysis was used to estimate the crude and adjusted odds ratio (OR) with 95% confidence intervals (95% CI) to investigate the relationship between responses to a hypothetical price increase and sociodemographic variables and smoking characteristics. All prevalence rate estimations and multivariate analysis were weighted to take into account different selection probabilities at each sampling stage. The Stata command “svy” was used to run the weights in all estimations.

## Results

Current smokers were more likely to be men and in the 20–29-year-old age group. Almost two thirds of the study population had attended school for less than nine years. Around half of the population smoked within the first 30 minutes after waking up. Almost 90% smoked up to 20 cigarettes per day. Regarding stages of change, smokers were mainly concentrated in the precontemplation stage followed by the contemplation and preparation stages (Table 1).

**Table 1:** Proportional distribution of expanded sample of current smokers (n = 500) by selected variables, 14 years or older, Brazil, 2005/2006

**Characteristics**	**Percentage**	**95% CI for percentage**
Sex		
Female	37.8	(32.9-42.8)
Male	62.2	(57.2-67.1)
Age group (years)		
14-19	5.3	(3.5-7.0)
20-39	46.5	(41.0-51.9)
40-59	36.4	(31.1-41.6)
60 or more	11.9	(8.5-15.3)
School level (years of schooling)		
Up to 9	71.3	(66.2-76.4)
9 or more	28.7	(23.6-33.8)
Employment status		
Unemployed	36.2	(30.8-41.5)
Employed	63.8	(58.5-69.2)
Time to first cigarette (TTFC)		
<30 min	46.3	(40.0-52.5)
≥30 min	53.7	(47.5-60.0)
Number of cigarettes per day		
>20	11.2	(8.1-14.3)
≤20	88.8	(85.7-91.9)
Stage of change		
Precontemplation	65.1	(59.3-70.8)
Contemplation	21.2	(16.8-25.5)
Preparation	13.8	(10.0-17.5)

In the full sample and in almost all the subgroups investigated, the most common participant response to price increases was, “I would try to stop smoking”. Their second choices were “I would smoke fewer cigarettes” and “I would look for a place where my brand is sold cheaper”, while “I would change to a cheaper brand” was the least frequently mentioned alternative (Table 2).

**Table 2:** **Percentages**^**(a) **^**of smokers in relation to what they said they would do in response to future price increases**^**(b)**^

	**Response involving quitting/smoking fewer cigarettes**		**Response involving price-minimizing strategies**	
	**Try to quit smoking**	**Smoke fewer cigarettes**	**Switch to a cheaper cigarette brand**	**Look for a cheaper source for their current cigarette brand**
Total	52.3	46.8	30.6	48.1
Sex				
Female	53.3	45.7	30.4	48.1
Male	51.7	47.5	30.7	48.1
p-value	0.76	0.76	0.96	1.00
Age group (years)				
14-19	55.3	61.5	46.1	60.8
20-39	52.8	49.2	29.8	47.9
40-59	54.0	43.2	27.4	48.9
60 or more	44.0	41.5	36.5	40.9
p-value	0.645	0.255	0.257	0.454
Schooling level (years)				
9 or more	**42.7**	**37.1**	**21.0**	**30.8**
Up to 9	**56.2**	**50.7**	**34.4**	**55.1**
p-value	**0.039**	**0.033**	**0.016**	**0.000**
Time to first cigarette (TTFC)				
<30 min	**42.2**	**37.5**	36.6	54.5
≥30 min	**60.7**	**54.6**	26.6	43.7
p-value	**0.002**	**0.004**	0.066	0.066
Number of cigarettes per day				
>20	42.3	**21.7**	39.4	60.1
≤20	53.5	**51.3**	29.8	47.1
p-value	0.17	**0.00**	0.25	0.13
Stage of change				
Precontemplation	**44.4**	41.9	31.1	47.0
Contemplation	**63.7**	55.2	25.9	45.5
Preparation	**72.2**	56.8	35.4	57.6
p-value	**0.002**	0.075	0.566	0.403

^(a)^Weighted percentages; ^(b)^The table only shows the percentage of individuals who answered "yes" to the non-mutually exclusive responses proposed. P-values from chi-square analysis. Entries in **bold** are statistically significant at p < 0.05 level.

The proportion of participants who reported that they would stop smoking as a result of a price increase was significantly higher among smokers with less than nine years of schooling. It was also significantly higher among those for whom the time to smoke first cigarette after waking up was greater than 30 minutes. There was a significant and progressive increase in the number of potential quitters from the precontemplation subgroup to the contemplation and preparation stage subgroups in response to a hypothetical price increase. Although not statistically significant, when compared with their counterparts, a greater proportion of participants who smoked fewer than 20 cigarettes per day reported that they would stop smoking. There were no differences in relation to sex or age (Table 2).

Regarding the estimates for the expectation of reducing the number of cigarettes in the hypothetical event of a price increase, the subgroup analysis was generally similar to the expectation of quitting smoking, although with lower percentages. The exception was found among 14–19-year-old smokers, for whom reducing the number of cigarettes in the event of an increase in price showed a higher proportion than the option of quitting smoking. In addition, the difference in the percentages between people who smoked up to 20 cigarettes per day in relation to those who smoked more than 20 cigarettes per day was statistically significant (Table 2).

The results shown in Table 2 indicate that “looking for a place that sells my brand cheaper” was mentioned more often than “changing to a cheaper brand”, in all subgroups investigated. Young people aged 14 to 19 years were the ones who most frequently indicated these two strategies for minimizing the effect of price increases as an alternative, when compared with other age groups. The highest percentages were also observed among individuals with fewer than nine years of schooling. Differences according to schooling level were statistically significant.

Multivariate analysis showed that statistically significant predictors of being potential quitters in the event of a hypothetical price increase were in the preparation stage and contemplation stage: time to smoke first cigarette after waking up greater than 30 minutes and lower schooling level. With the exception of the preparation stage of change, these variables were also predictors that individuals would say that they would react by smoking less cigarettes. However, differently from what was observed in individuals saying that they would stop smoking, the groups that presented the strongest association were the 14–19-year-old teenagers and individuals smoking up to 20 cigarettes per day (Table 3).

**Table 3:** **Predictors for hypothetical reactions to higher prices among current smokers that may have an impact on reducing tobacco smoking**^**(a)**^

**Individual characteristics**	**Try to quit smoking**		**Smoke fewer cigarettes**	
	**OR**	**(95% CI for OR)**	**OR**	**(95% CI for OR)**
Sex				
Female	1		1	
Male	0.76	(0.47-1.22)	0.71	(0.42-1.21)
Age group (years)				
20 or more	1		1	
14 - 19	1.54	(0.70-3.41)	**3.44**	**(1.74-6.77)**
Schooling level (years)				
9 or more	1		1	
Up to 9	**2.04**	**(1.14-3.63)**	**2.48**	**(1.41-4.34)**
Time to first cigarette (TTFC)				
<30 min	1		1	
≥30 min	**2.19**	**(1.28-3.73)**	**1.74**	**(1.01-3.01)**
Number of cigarettes per day				
>20	1		1	
≤20	1.17	(0.58-2.35)	**3.36**	**(1.48-7.63)**
Stage of change				
Precontemplation	1		1	
Contemplation	**2.46**	**(1.22-4.95)**	**1.99**	**(1.06-3.75)**
Preparation	**2.97**	**(1.35-6.54)**	1.43	(0.64-3.20)

^(a)^Multivariate logistic regression model assessing whether the demographic and smoking characteristics of interest were predictive of reported responses to hypothetical price increase. Each variable was adjusted simultaneously for all other variables shown in the table. Entries in bold are statistically significant at p < 0.05 level.

Lower schooling level was the only statistically significant positive predictor, both for the response that the individual would change to a cheaper brand and for the response that the individual would look for a place that sold their preferred brand more cheaply. Being young also presented a positive association, although this was not significant (Table 4).

**Table 4:** **Predictors for hypothetical reactions to higher prices among current smokers that may minimize the impact of cigarette price increases**^**(a)**^

**Individual characteristics**	**Switch to a cheaper cigarette brand**		**Look for a cheaper source for their current cigarette brand**	
	**OR**	**(95% CI for OR)**	**OR**	**(95% CI for OR)**
Sex				
Female	1		1	
Male	1.00	(0.61-1.63)	0.97	(0.62-1.52)
Age group (years)				
20 or more	1		1	
14 - 19	2.07	(0.92-4.66)	1.93	(0.86-4.35)
Schooling level (years)				
9 or more	1		1	
Up to 9	**2.02**	**(1.15-3.56)**	**2.79**	**(1.64-4.73)**
Time to first cigarette (TTFC)				
<30 min	1		1	
≥30 min	0.69	(0.41-1.16)	0.74	(0.46-1.19)
Number of cigarettes per day				
>20	1		1	
≤20	0.77	(0.35-1.67)	0.67	(0.33-1.37)
Stage of change				
Precontemplation	1		1	
Contemplation	0.75	(0.39-1.45)	0.92	(0.51-1.66)
Preparation	1.24	(0.59-2.61)	1.62	(0.85-3.10)

^(a)^Multivariate logistic regression model assessing whether the demographic and smoking characteristics of interest were predictive of reported responses to hypothetical price increase. Each variable was adjusted simultaneously for all other variables shown in the table. Entries in bold are statistically significant at p < 0.05 level.

## Discussion

This is possibly one of the first studies conducted in Latin American that examined smokers’ responses to a hypothetical cigarette price increase, and to compare the responses to reduce consumption with that aimed to reduce the expenditure on cigarettes while maintaining their consumption level. Furthermore, in the English literature, few studies with similar objectives were conducted previously, all in developed countries [24-26]. The results presented here are especially important because cigarette prices in Brazil will be increased by 50 centavos per year, starting in 2014. This is still a modest increase, and will require further attention to ensure that it impacts smoking consumption properly [13].

The present study suggests that smokers’ preferred choice for facing a hypothetical price increase would be to quit, and that other alternatives could minimize the cessation effect of prices increases, but would not neutralize this effect. This finding is consonant with results in a previous study conducted in Brazil and in another 19 countries, when smokers were asked to give their reasons to think about stopping smoking [27]. Together with the United States of America, Brazil was the first in the ranking with 74.4% of the smokers responding that the price would be a reason for stopping smoking.

Given that the intention of quitting has been shown as an important predictor of smoking cessation, the findings from the present study reinforce the expectation that a progressive taxes-and-prices policy has great potential for reducing tobacco consumption and smoking prevalence in Brazil [22,28]. These results are particularly important, considering that this is one of the few effective tobacco control measures that is not developed at its full potential in the country [13].

The high prevalence rates of smokers who responded that they would look for a place that would sell the same brand they usually smoke but cheaper is a matter for concern. Since the price of different brands of cigarettes follows a prescribed price table within each state, the only alternative for obtaining cigarette brands at lower prices is the illegal market, especially through street-sellers, scattered across the country. In fact, during the 1990s, a tendency towards an increased market share for illegal products, was observed after an increase in cigarette prices [15]. However, the results from the present study suggest that seeking illegal products may reduce the impact of increased taxes, but does not eliminate it [3]. Studies in Brazil suggest that increasing the real price of cigarettes has historically led to reductions in total consumption, despite the presence of an illegal market [14,15].

Regarding the observed percentages of smokers who reported that they would change to a cheaper brand, this result was similar to what was found in a study conducted in California among around 5,000 smokers [9]. A series of cross-sectional studies conducted in Germany showed that although this was a less frequently mentioned option among smokers, in the event of a hypothetical price increase, the percentage of smokers who actually used this strategy to adjust their budgets was more evident after the price increase [25].

It was noteworthy that after controlling for all other variables studied, having a low schooling level was the only variable that remained as a predictor for all the possible hypothetical responses to cigarette price increases, irrespective of whether the responses related to reduced consumption or to alternatives that might minimize the impact of the prices increases. Studies have shown that poor smokers with low schooling levels respond more positively to taxes and prices increases if the illegal market was brought under proper control [2]. This is also the subgroup with the greatest likelihood of buying cigarettes in the illegal market, which is in line with the findings of a high percentage mentioning that they would “look for a place that sells the brand smoked with cheaper prices”. This shows the importance of measures to combat the illegal market that can undermine the effectiveness of tax and prices policies. The illegal trade protocol of the WHO Framework Convention on Tobacco Control was recently adopted and Brazil will certainly benefit from becoming a Party to the Protocol [29].

Both in uni- and multivariate analyses, individuals with lower levels of dependence were more likely to say that they would respond to a hypothetical price increase by trying to stop smoking or even by reducing the number of cigarettes when compared with individuals with greater levels of dependence. A stronger association with attempts to quit smoking was found. The degree of nicotine dependence is inversely related to attempts to stop smoking [28,30]. A previous study also observed that people who smoke fewer cigarettes per day are more likely to affirm that they would respond by quitting and/or reducing consumption when compared with those who smoke more cigarettes per day [24]. However, in the present study, smoking a smaller number of cigarettes per day was only a predictor for smokers mentioning that they would reduce the number of cigarettes smoked per day.

It is worth noting that after controlling for stages of change for cessation, the population subgroups that had the greatest likelihood of saying that they would respond to price increases by considering stopping smoking or by diminishing the number of cigarettes (i.e., individuals who presented lower dependence levels and those with lower schooling levels) are the same as the largest proportion of smokers in Brazil. Based on analysis of the Heaviness of Smoking Index among participants in the Global Adult Tobacco Survey (conducted in 2009), 81.3% of Brazilian smokers had low-to-moderate dependence [31]. Given also that the greatest proportion of this subgroup belongs to the lower socioeconomic level strata, it can be supposed that many low-income smokers will try to stop smoking after cigarette taxes and prices have been increased, which will have a double health and economic benefit.

Regarding the analysis according to age group, it should be highlighted that younger smokers did not seem to be the subgroup that would respond to taxes and prices increase policies with cessation. However, being young (14–19 years) was the main independent predictor for individuals to say that they would smoke fewer cigarettes. Despite a lack of statistical significance, an association with alternative responses to the hypothetical price increase that would minimize the impact of this measure was also observed. These findings are supported by the literature. A recent review on the effectiveness of taxes and prices policies for controlling smoking showed that there was sufficient evidence that such policies reduced the consumption among young people, but that there was no evidence to show that this subgroup would stop smoking through this policy. Nevertheless, these studies showed that taxes and prices increases would prevent conversion of young experimenters into regular smokers who might then become dependent on nicotine, through reducing accessibility, given the low purchasing power of this age group [2,3].

As a cross-sectional study conducted before tax and price increases, potential future reactions were estimated. We are aware of the inherit limitation of this approach and the data will neither necessarily reflect actual reactions nor independent choices. Nonetheless, given the lack of any other data in Brazil estimating smokers’ reactions to price changes, we believe that the study will contribute to the body of knowledge and guide a future longitudinal study.

## Conclusions

The present study concludes that tax and price increases have great potential to further stimulate smoking cessation in Brazil. It was more evident that the population subgroup with the greatest prevalence of smokers (those belonging to the less-educated social classes) may react to such measures in two ways: by reducing their consumption or by seeking cheaper products in the illegal cigarette market. To deal with this situation, the country should consider: (a) further strengthening tax and pricing policies; (b) expand the provision of free-of-charge treatment for nicotine dependence and invest in training programs for health care professionals in relation to brief interventions and treatments for smokers with lower degrees of nicotine dependence; and (c) strengthen policies to control the illegal cigarette market including becoming a Party to the WHO Framework Convention on Tobacco Control Illicit Trade Protocol.

## Competing interests

The authors declare that they have no competing interests.

## Author’s contributions

APG planed the article and participated in the design, data analysis, drafting, and final editing of the article. VCF participated in the design, data analysis, drafting, and final editing of the article. CSM substantially contributed to the data analyses and article revision. VLdaCS substantially contributed in drafting and final editing of the article. RC, ACPRM, IP and MR substantially contributed to the final editing of the article and RL participated in the conception of this project and collaborated in the design, drafting, and final revision of the manuscript. All authors read and approved the final manuscript.

## Pre-publication history

The pre-publication history for this paper can be accessed here:

http://www.biomedcentral.com/1471-2458/14/327/prepub
